# Case of Congenital Tularemia with Neuroinvasive Disease, Utah, USA

**DOI:** 10.3201/eid3112.250703

**Published:** 2025-12

**Authors:** Brent D. Nelson, Amara Finch, Krow Ampofo, Elizabeth L. Ryals, Andrew T. Pavia, Anne J. Blaschke, Jody L. Lin, Benjamin Kalm, Angie White, Kacy D. Nowak, Julian A. Villalba, Julu Bhatnagar, Bert Lopansri, Elizabeth D. Knackstedt

**Affiliations:** University of Utah School of Medicine, Salt Lake City, Utah, USA (B.D. Nelson, A. Finch, K. Ampofo, A.T. Pavia, A.J. Blaschke, J.L. Lin, B. Kalm, B. Lopansri, E.D. Knackstedt); Primary Children’s Hospital, Salt Lake City (E.L. Ryals); Bear River Health Department, Logan, Utah, USA (A. White); Utah Department of Health and Human Services, Salt Lake City (K.D. Nowak); Centers for Disease Control and Prevention, Atlanta, Georgia, USA (J.A. Villalba, J. Bhatnagar); Intermountain Medical Center, Murray, Utah, USA (B. Lopansri)

**Keywords:** tularemia, bacteria, gram-negative bacterial infections, congenital infection, *Francisella tularensis*, zoonoses, brain abscess, meningitis/encephalitis, Utah, USA

## Abstract

We diagnosed neuroinvasive tularemia in a neonate in Utah who had culture-negative pleocytosis in cerebrospinal fluid, rim-enhancing lesions on brain magnetic resonance imaging, and blood microbial cell-free DNA *Francisella tularensis* detection. Maternal history, serologic testing, and *Francisella* sp. identified in the fallopian tube by immunohistochemistry and 16S rRNA gene PCR strongly support congenital infection.

Tularemia, caused by *Francisella tularensis*, is a bacterial illness endemic to the Northern Hemisphere (*1*). Tularemia classically presents as ulceroglandular, glandular, oculoglandular, oropharyngeal, pneumonic, or typhoidal disease; other manifestations have been described (*1*–*4*). Neuroinvasive disease, although rare and difficult to diagnose, has also been reported (*5*–*7*).

Rarely, vertically transmitted tularemia in animals with histopathological and immunohistochemical (IHC) confirmation of *F. tularensis* in aborted fetuses (*8*,*9*) has been reported. One presumed case of human congenital infection has been reported. In 1947, Lide (*10*) reported delivery of a stillborn infant after tularemia was diagnosed in the mother. Gram-negative bacilli were observed in placental and fetal tissues without confirmatory testing (*10*).

We recently diagnosed congenital, neuroinvasive tularemia in a neonate after a positive blood test for microbial cell-free DNA (cfDNA) (Karius, https://kariusdx.com). IHC staining and 16S rRNA gene PCR identified *F. tularensis* in the mother’s fallopian tube.

## The Study

**Figure 1:**
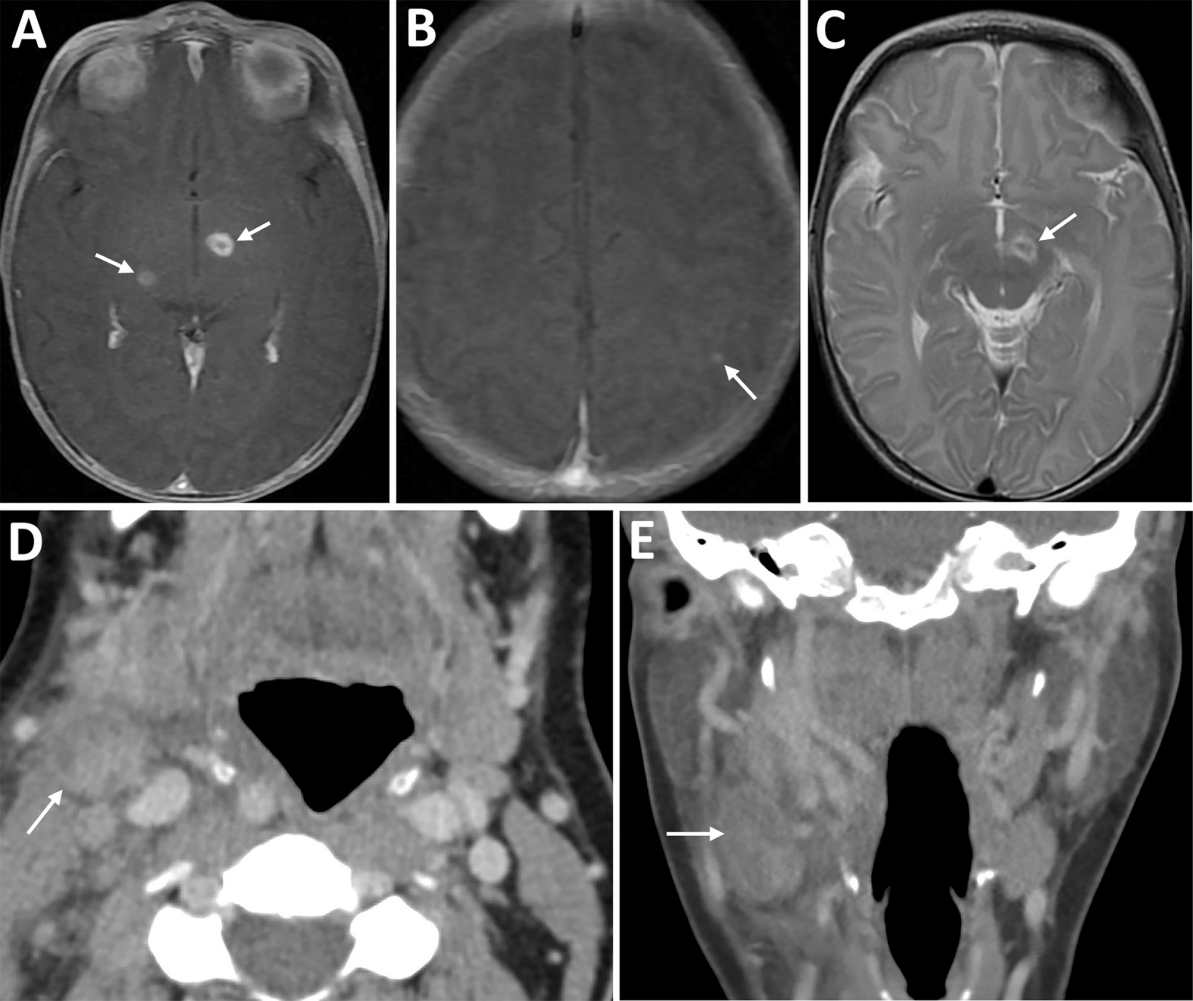
Imaging from infant and mother in case of congenital tularemia with neuroinvasive disease, Utah, USA. A, B) Axial T1 post-contrast images showing the infant’s initial magnetic resonance imaging findings of rim enhancing lesions near the left subthalamic nucleus and right inferior thalamus (arrows, panel A), as well as a punctate enhancing lesion in the left parietal lobe (arrow, panel B). C) Axial T2 image demonstrating T2 hyperintense edema along the margins of the largest lesion near the left subthalamic nucleus (arrow). D, E) Axial (D) and coronal (E) images from the mother’s computed tomography scan with intravenous contrast showing an enlarged, heterogeneous right cervical chain lymph node with inflammatory stranding in the adjacent soft tissues (arrows).

A 2-week-old infant was admitted to a hospital in Salt Lake City, Utah, USA, with lethargy, poor feeding, and pallor. The mother had ulcerative colitis in remission on infliximab therapy. One week after her infliximab dose at 34 weeks’ gestation, she experienced fever, sore throat, conjunctivitis, and cervical lymphadenopathy. After 6 days, a computed tomography scan of the neck revealed cervical lymphadenitis (Figure 1). An otolaryngologist evaluated and treated her with ceftriaxone and dexamethasone. She was evaluated by obstetrics 4 days later for decreased fetal movement. A biophysical profile score of 8 was reassuring. She underwent incision and drainage of her cervical lymph node. During the procedure, purulent fluid was encountered and sent for routine and acid-fast bacilli culture; no growth was noted at 4 days on routine culture or 42 days on acid-fast bacilli culture. She completed 10 days of amoxicillin/clavulanate.

The infant was born at 37 weeks’ gestation by cesarean section because of gestational hypertension and ongoing maternal illness. At delivery, the infant required 15 minutes of respiratory support but weaned to room air and was discharged home on day of life (DOL) 2. On DOL 16, he was seen in the emergency department for lethargy and decreased oral intake. He was tachycardic but afebrile. Complete blood count, complete metabolic panel, C-reactive protein, procalcitonin, and blood, urine, and cerebral spinal fluid (CSF) cultures were obtained; results were notable for marked peripheral leukocytosis, elevated C-reactive protein, hepatitis, and lymphocytic CSF pleocytosis (Table 1). The infant received ampicillin and ceftazidime.

**Table 1:** Pertinent results of laboratory tests taken during hospitalization of infant in case of congenital tularemia with neuroinvasive disease, Utah, USA*

Test	Hospitalization 1			Hospitalization 2
	Admission: DOL 16	Discharge: DOL 21		Admission: DOL 30
Complete blood count				
Hemoglobin, K/μL	13.8	12.6		13.1
Hematocrit, g/dL	39.8	**36.7**		39.6
Leukocyte, K/μL	**56.8**	17.6		**25.2**
Neutrophils, K/μL	**29.0**	3.3		**4.8**
Lymphocytes, K/μL	**19.9**	11.3		**15.4**
Monocytes, K/μL	**4.5**	**3.0**		**3.5**
Platelets, K/μL	206	**149**		**584**
Chemistry				
Sodium, mmol/L	**129**	144		137
Total bilirubin, mg/dL	1.3	1.1		0.9
ALP, unit/L	205	139		335
AST, unit/L	**898**	**144**		57
ALT, unit/L	**316**	**96**		26
Lactic acid, mmol/L	**2.4**			1.8
Inflammatory/infectious markers				
C-reactive protein, mg/dL	**17.1**	**6.8**		**4.7**
Procalcitonin, ng/mL	**3.02**			**0.15**
Cerebrospinal fluid				
Glucose, mg/dL	44	NA		**36**
Total protein, mg/dL	**114**	NA		81
Leukocytes, cells/μL	**48**	NA		**31**
Neutrophils, %	3	NA		4
Lymphocytes, %	81	NA		71
Monocytes, %	16	NA		25
Erythrocytes, cells/μL	<1	NA		**7**
Gram stain	Negative	NA		Negative
BioFire Respiratory 2.1 panel	**Positive for rhinovirus**			**Positive for rhinovirus**
Cultures				
Blood	No growth × 5 d	NA		No growth × 5 d
Urine	1,000 colony-forming units of *Staphylococcus lugdunensis*	NA		No growth × 2 d
CSF	No growth × 4 d	NA		No growth × 4 d

*Abnormal values in bold. ALP, alkaline phosphatase; ALT, alanine transaminase; AST, aspartate aminotransferase; DOL, day of life; NA, not applicable.

Increasing lethargy and new oxygen requirement prompted transfer to the pediatric intensive care unit. At arrival, he was febrile to 39.6°C. Results of blood PCR testing for adenovirus, parvovirus B19, and cytomegalovirus were negative. A multiplex PCR panel (BioFire, https://www.biofiredx.com) detected human rhinovirus/enterovirus on a nasopharyngeal swab specimen. Results of multiplex PCR testing of the CSF (BioFire Filmarray Meningitis/Encephalitis Panel) were negative. During hospitalization, the infant developed thrombocytopenia to 115 K/μL. Bacterial blood cultures remained negative, and laboratory abnormalities improved (Table 1). He was observed for 24 hours off antibiotics, then discharged home.

He returned to the emergency department 9 days later, DOL 30. Vital signs were unremarkable, although he appeared unwell. He was transferred to our institution for further evaluation and infectious disease consultation.

Repeat lumbar puncture showed persistent lymphocytic CSF pleocytosis, and he underwent brain magnetic resonance imaging (MRI) with contrast. The MRI revealed 3 lesions, 2–7 mm in diameter, located in the left subthalamic nucleus, right thalamus, and left parietal cortex (Figure 1). Results of PCR testing of the CSF for toxoplasmosis were negative. Because of persistent illness and previously nondiagnostic evaluation, blood cfDNA testing (Karius) was performed, and results were positive for 804 molecules of microbial cfDNA/μL of *F. tularensis* DNA most aligned with subspecies *holartica* (Table 2). Treatment with intravenous (IV) ciprofloxacin and gentamicin was initiated.

**Table 2:** Infant and maternal testing for *Francisella tularensis* in study of congenital tularemia with neuroinvasive disease, Utah, USA*

**Test, specimen source**	**Infant**	**Mother**
**Microbial cell free DNA metagenomic sequencing (Karius), plasma†**	4 weeks: 804 MPM *F. tularensis* subsp. *holartica* cfDNA (Appendix)	NA
	7 weeks: 11 MPM *F. tularensis subsp. holartica* cfDNA (Appendix)	
***F. tularensis* IgM, serum**	4 weeks: negative	6 weeks postpartum: positive
	8 weeks: negative	
***F. tularensis* IgG, serum**	4 weeks: positive	6 weeks postpartum: positive
	8 weeks: negative	
**Culture data**	(Table 1)	Neck abscess (35 weeks’ gestation): no growth × 5 d
		Throat culture (6 weeks postpartum): no growth × 5 d
**Next-generation metagenomic sequencing, CSF‡**	4 weeks: negative	NA
**Multiplex PCR panel (BioFire), serum**	4 weeks: negative	NA
**Multiplex PCR panel, (BioFire); meningitis/encephalitis panel (Filmarray), CSF**	4 weeks: negative	NA
***Francisella tularensis* immunohistochemistry, FFPE maternal fallopian tube tissue§**	NA	*Francisella* spp. antigens detected (Figure 2)
**Gram-negative bacteria 16S rRNA gene PCR test, FFPE maternal fallopian tube tissue§**	NA	Positive for *Francisella* spp.

*cfDNA, cell-free DNA; CSF, cerebrospinal fluid; FFPE, formalin-fixed paraffin-embedded; MPM, molecules per microliter; NA, not applicable. **†Testing performed at Karius (https://kariusdx.com).** **‡Testing performed at University of California, San Francisco (San Francisco, CA, USA).** **§Testing performed at Centers for Disease Control and Prevention (Atlanta, GA, USA).**

The family lives on a multiple-acre property supplied by well water with nearby irrigation canals and a beaver population. Pets consisted of 1 rabbit, 2 hunting dogs, and a cat. The family had bred rabbits until ≈2 years before. The cat hunted mice and voles and was frequently in close physical contact with the mother. The cat had been “vomiting up worms” and, 2 days before the infant’s second hospitalization, was run over by a tractor and died.

On day 40 postpartum, the mother was seen by an infectious disease physician because of the infant’s tularemia diagnosis. She had continued to experience night sweats, fatigue, and anorexia, and new, diffuse arthralgias had developed, most prominently in her hands. Throat culture results were negative, but tests for *F. tularensis* IgG and IgM were positive (Table 2). She received 14 days of ciprofloxacin and subsequently returned to her baseline state of health.

The county health department investigated the home. The well water had increased coliform counts, but well water PCR test results for *F. tularensis* were negative. All other family members tested negative for *F. tularensis* antibodies. Their rabbit had been euthanized and was unavailable for further testing, as was the cat.

The infant received 1 week of IV gentamicin and 4 weeks of IV ciprofloxacin. Near the end of therapy, repeat microbial cfDNA testing (Karius) demonstrated a marked decline in *F. tularensis* microbial cfDNA levels (Table 2). Repeat brain MRI with contrast showed near complete resolution of the previous lesions with only “trace residual focus of enhancement in the left subthalamic region.” He remains well, last evaluated at 15 months of age.

**Figure 2:**
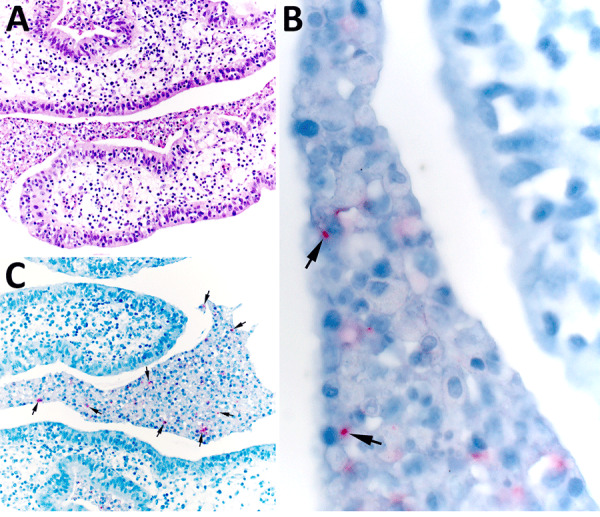
Histopathology in study of congenital tularemia with neuroinvasive disease, Utah, USA. Histopathological evaluation revealed the presence of subacute suppurative salpingitis with serositis. A) Hematoxylin and eosin–stained tissue showing abundant necroinflammatory debris in the lumen of the left fallopian tube. The endosalpinx was edematous, and infiltrating neutrophils and mononuclear cells were seen in the lamina propria and tubal epithelium. Original magnification ×200. B) Higher-power microphotograph highlights immunostaining within intracellular coccobacilli (arrows). Original magnification ×630. C) *Francisella tularensis* immunohistochemistry showing immunoreactive granular forms of bacterial antigens (arrows) within areas of the luminal necroinflammatory infiltrate. Original magnification ×200.

The mother underwent elective tubal ligation during her cesarean section. Extensive left-sided subacute suppurative salpingitis with serositis incidentally was noted. Once the diagnosis of congenital tularemia was suspected, remaining formalin-fixed paraffin-embedded tissue were sent to the Centers for Disease Control and Prevention, where results of an IHC assay for *F. tularensis* were positive for bacterial antigens with coccobacilli in the left fallopian tube, along with necroinflammatory debris (Figure 2) (*11*). In addition, a gram-negative bacteria 16S rRNA gene PCR performed on DNA extracts from a formalin-fixed paraffin-embedded tissue block containing tissue of the left fallopian tube was positive for *Francisella* spp.

## Conclusions

We report vertical transmission of tularemia, resulting in congenital infection with neuroinvasive disease in a neonate. The findings of CSF lymphocytic pleocytosis, 3 discrete brain lesions, and positive blood cfDNA testing for *F. tularensis* on 2 separate samples, with resolution of brain lesions after therapy, support this diagnosis. The mother’s illness at 34 weeks’ gestation was consistent with oropharyngeal tularemia and supported by serologic testing 40 days postpartum. The detection of *F. tularensis* and 16S rRNA gene by PCR in the fallopian tube confirm tularemia-induced salpingitis and presumed vertical transmission.

Neuroinvasive tularemia is uncommon; lymphocytic meningitis is the most common manifestation. Rare reports exist of discrete brain lesions (*5*). Various antibiotic medications have been described in treatment of neuroinvasive tularemia, such as streptomycin, gentamicin, doxycycline, chloramphenicol, and ciprofloxacin, often in combination (*5*).

This case of vertical transmission of *F. tularensis* in humans, supported with microbiological confirmation by histopathological and molecular methods, is unique. Complications of tularemia in pregnancy have been reported previously (*10*,*12*–*14*). The source of maternal infection remains unclear. A concurrent zoonotic outbreak of tularemia among beavers occurred in neighboring counties, and the mother had multiple other potential exposures. However, we found no clear linkage (*15*).

This case highlights the possibility of vertical transmission of tularemia, as well as neurologic manifestations in neonates. Diagnosis can be challenging and require assistance from state health departments and specialized commercial and Centers for Disease Control and Prevention national reference laboratories.

Appendix Additional information about case of congenital tularemia with neuroinvasive disease, Utah, USA
