# Supplementary material for: Case of Congenital Tularemia with Neuroinvasive Disease, Utah, USA
Source: Emerg Infect Dis. 2025 Dec;31(12):2284–8. doi: 10.3201/eid3112.250703 (PMC12782186; doi:10.3201/eid3112.250703)
Supplement: Appendix — Additional information about case of congenital tularemia with neuroinvasive disease, Utah, USA [file 25-0703-Techapp-s1.pdf]

Article DOI: https://doi.org/10.3201/eid3112.250703

EID cannot ensure accessibility for supplementary materials supplied by authors. Readers who have difficulty accessing supplementary content should contact the authors for assistance.

Case of Congenital Tularemia with Neuroinvasive Disease, Utah, USA

Appendix

Appendix Table. BLAST alignment of mcfDNA fragments supports identification of *Francisella tularensis*. Microbial cell-free DNA fragments were processed from a neonatal sample at 4 weeks and sequenced as described previously (*1*). After removal of human and spiked-in sequences, short read sequences were aligned to whole-genome assemblies of *F. tularensis* and the closely related species *F. hispaniensis*. The mean BLAST percent nonidentity (100 - percent identity) and the number of reads aligned with at least 80% identity are shown for one assembly of *F. hispaniensis* and up to 3 assemblies of each subspecies of *F. tularensis* having the highest percent identity. Both measures strongly support the identification of *F. tularensis* at the species rank. In addition, these measures are consistent with *F. tularensis* subspecies *holarctica* (type B) over other subspecies, including *F. tularensis* subspecies *tularensis (type A)* and *F. tularensis* subspecies *novicida (F. novicida).*

species	subspecies	100% - mean BLAST % identity	mean BLAST % identity	BLAST aligned short reads	assembly accession
Francisella tularensis	*holarctica*	0.25	99.75	415	GCA_019134795.1
Francisella tularensis	*holarctica*	0.25	99.75	417	GCA_000011405.1
Francisella tularensis	*holarctica*	0.25	99.75	417	GCA_000014605.1
Francisella tularensis	*tularensis*	0.54	99.46	399	GCA_000305835.1
Francisella tularensis	*tularensis*	0.54	99.46	398	GCA_000305895.1
Francisella tularensis	*tularensis*	0.54	99.46	398	GCA_000305915.1
Francisella tularensis	*mediasiatica*	0.71	99.29	406	GCA_000018925.1
Francisella tularensis	*mediasiatica*	0.71	99.29	406	GCA_016604655.1
Francisella tularensis	*mediasiatica*	0.71	99.29	406	GCA_016604685.1
Francisella tularensis	*novicida*	1.45	98.55	382	GCA_001865695.1
Francisella tularensis	*novicida*	1.48	98.52	386	GCA_016604535.1
Francisella tularensis	*novicida*	1.49	98.51	377	GCA_016604325.1
Francisella hispaniensis		6.68	93.32	296	GCA_001885235.1

Reference

1. Blauwkamp TA, Thair S, Rosen MJ, Blair L, Lindner MS, Vilfan ID, et al. Analytical and clinical validation of a microbial cell-free DNA sequencing test for infectious disease. Nat Microbiol. 2019;4:663–74. PubMed https://doi.org/10.1038/s41564-018-0349-6
